# Exploring Complementary Medicine Usage, Consumer Perceptions, and Impact of Label Warnings: A Cross-Sectional Study in Melbourne, Australia

**DOI:** 10.3390/pharmacy13030061

**Published:** 2025-04-27

**Authors:** Kaveh Naseri, Thilini Thrimawithana, Ayman Allahham, Vivek Nooney, Barbora de Courten, Wejdan Shahin

**Affiliations:** Discipline of Pharmacy, School of Health and Biomedical Sciences, RMIT University, Bundoora, VIC 3083, Australia; s3961858@student.rmit.edu.au (K.N.); thilini.thrimawithana@rmit.edu.au (T.T.); ayman.allahham@rmit.edu.au (A.A.); vivek.nooney@rmit.edu.au (V.N.); barbora.decourten@rmit.edu.au (B.d.C.)

**Keywords:** complementary medicines, consumer perceptions, label warning, adverse reactions

## Abstract

Complementary medicines (CMs) are widely used worldwide, with usage rates ranging from 24% to 71.3%. Despite their popularity, many CMs lack robust scientific support and can potentially lead to adverse health effects. Limited research exists on CMs-related adverse events and the role of CMs’ labels in conveying crucial information to consumers. This cross-sectional study investigated the usage, consumer perspectives, and influence of labels specifically on product-based CMs, including nutritional supplements, vitamins, minerals, probiotics, prebiotics, and herbal medicines. Practitioner-led therapies and mind-body practices were outside the scope of this research. Data were collected through an online questionnaire and analyzed using descriptive statistics and correlation analysis. The study enrolled 125 participants who were current CMs users. Pharmacies and supermarkets were the primary sources for CMs procurement. Participants’ perceptions of CMs effectiveness and safety were positively correlated. Label warnings prompted participants to seek additional information, but consultation with healthcare professionals was infrequent. Adverse reactions were reported by 18.5% of participants, with self-management approaches being common. Label warnings play a significant role in prompting consumers to seek more information about CMs. However, the limited engagement of healthcare professionals, especially pharmacists, suggests an opportunity for improved consumer education and pharmacist involvement in CMs-related discussions. Addressing these aspects can lead to safer CMs practices and informed decision-making among consumers.

## 1. Introduction

Worldwide, the utilization of complementary medicines varies significantly, with usage rates ranging from 24% to 71.3% [1]. In this study, CMs are defined as medicinal products, such as vitamins, minerals, herbal preparations, and nutritional supplements, and exclude practitioner-led therapies (e.g., acupuncture, chiropractic care) and mind-body practices. A recent cross-sectional study conducted in Australia reported that 50.3% of the study participants (n = 2019) used CMs, with the majority of the population using vitamin/mineral supplements (47.8%) [2]. It is important to note that women are more likely to use CMs than men. Moreover, Australians with a chronic disease diagnosis are also more likely to use CMs compared to the general population.

Community pharmacies play a substantial role in offering a wide array of CMs to consumers [3]. Community pharmacists are commonly regarded as the primary link between patients and health care systems. Being strategically positioned within the community allows them to be involved in a broad range of health promotion campaigns and services in addition to promoting safe and effective CMs use [4]. While there is evidence supporting an increased role for pharmacists in the realm of CMs utilization, the specific levels and types of pharmaceutical care that community pharmacists should offer concerning these products remain unclear [5]. This ambiguity is particularly concerning given that many CMs lack rigorous research to support their use and may cause harm if not used appropriately [6].

The utilization of certain CMs in disease treatment and prevention has raised concerns among healthcare professionals and researchers. These concerns arise from limited scientific evidence supporting their efficacy, the potential for adverse effects, possible interactions with conventional medicines, and the widespread use of CMs, which often stems from the perception that these products are inherently safe [5]. Nonetheless, this belief lacks robust support from emerging data, which increasingly reveals the actual prevalence of adverse reactions and drug interactions associated with the use of CMs [5]. Numerous studies have consistently underscored the potential for serious toxicities and adverse reactions linked to the use of CM products [7]. However, research on CMs-related adverse events in Australia is limited.

A study by the National Prescribing Service found that consumers often perceive complementary medicines as safer alternatives to conventional medications, yet they may not be fully informed about the potential risks associated with them, including side effects, toxicity, the possibility of allergic reactions, and interactions with conventional medication [8].

A study conducted in the United States of America emphasized the importance of sharing research findings and safety concerns regarding CMs with both healthcare professionals and the public [9]. The research underscores a prevalent misconception among consumers who believe CM therapies and dietary supplements are inherently safe due to their natural ingredients, cautioning that they can pose both direct and indirect harm [9]. Furthermore, previous research has highlighted the potential health risks of CMs in cancer patients, emphasizing the importance of healthcare professional consultations prior to CM purchases [10].

Moreover, a Malaysian study reported that 26% of CM users experienced adverse events [11], whereas a study conducted in the United Kingdom identified 45.8% of consumers reporting adverse events related to CM use [12]. Underreporting of CMs-related adverse events is common. Braun et al. found that only 19% of participants reported adverse events [13], and Barnes et al. highlighted differences in reporting adverse events between CMs and non-CMs [14]. Factors contributing to this variation in adverse events across different parts of the world include perceptions of CMs safety, unsupervised usage, and limited awareness of reporting requirements [15].

In Australia, the Therapeutic Goods Administration (TGA) oversees the monitoring of the safety of medicines and relies on the reporting of adverse events to improve patient safety. While it is mandatory for pharmaceutical companies to report all serious adverse reactions suspected of being related to their medicines, reporting by health professionals has traditionally been voluntary. Health professionals and consumers can report adverse events of medicines to the TGA via email, fax, mail, or online forms [16].

The Expert Committee on Complementary Medicine in Australia raised several concerns regarding the safe, appropriate, and effective use of CMs. Among these concerns was the necessity for both consumers and health professionals to access accurate, reliable, and independent information about CMs. Additionally, there was a call for individuals to develop appropriate skills to interpret available information and discern between reliable and unreliable sources to allow healthcare professionals to make informed decisions about the use of CMs [8].

Labels provide crucial information about these medicines, including the strength of the active ingredients, excipient details, dosage, and safety warnings [17]. Evaluating the list of ingredients is essential in clinical decision-making and in considering the risks and benefits. Previous studies have primarily focused on the popularity, efficacy, adverse events, and consumer perspectives of CMs [10,18,19,20,21]. However, there is limited literature on consumer perception or the use of CMs labels. The specific attention given to labels as conduits for providing information and guiding consumers’ choices remains relatively understudied. This gap in the literature leaves unanswered questions about how effectively labels convey essential information about CMs for consumers, including details about ingredients, potential interactions, and adverse events, as well as the subsequent reactions of consumers to this information.

Hence, this study aimed to investigate CMs utilization in Melbourne, Australia, as well as consumers’ views on their effectiveness and adverse events. Moreover, it seeks to assess how labels influence CM usage, especially in terms of consumer comprehension, seeking more information, and consulting with healthcare professionals. In this study, CMs refer specifically to products, such as nutritional supplements, vitamins, minerals, probiotics, prebiotics, and herbal medicines that are sold over the counter in pharmacies and supermarkets. The scope of this study does not encompass practitioner-led therapies (e.g., acupuncture, chiropractic care, or massage) or mind-body practices. Therefore, the findings presented herein are limited to product-based CMs.

## 2. Materials and Methods

### 2.1. Ethics Approval

The study received ethics approval from the RMIT University Ethics Committee under project number 25543.

### 2.2. Study Design and Setting

A cross-sectional design was employed to gather data on participants’ attitudes and practices towards CMs. To ensure an adequate sample size, recruitment was initiated through a multi-faceted approach. Participants were recruited over a two-week period from 4 to 18 September 2022. Initially, recruitment efforts were launched on social media platforms, including on the RMIT pharmacy X group (a X group managed by the members of the Pharmacy department at RMIT University, used as a platform for sharing research-related updates and student activities). Subsequently, project flyers were distributed, featuring a QR code and an online survey link. A 20 AUD voucher incentive for one in five participants was prominently displayed on the flyers to encourage participation. These flyers were disseminated at various community pharmacies across Melbourne, Australia. Furthermore, a direct survey link was shared within personal and professional networks to enhance the diversity of the sample. Participants received an invitation letter that provided detailed information about the survey’s objectives, estimated completion time, and the advisory committee overseeing the project.

### 2.3. Study Participants

Inclusion criteria for participation required individuals to be at least 18 years old, users (within the past six months) of product-based CMs available over the counter (e.g., vitamins, minerals, and herbal preparations), currently living in Melbourne, Australia, and proficient in English to understand and complete the questionnaire. Participants were informed that by completion of the questionnaire, they were giving their informed consent to participate. Potential participants under the age of 18, those who had not used CMs within the past 6 months, non-residents of Melbourne, Australia, individuals lacking English proficiency, and those who did not provide informed consent were automatically excluded from the survey to ensure relevancy.

### 2.4. Development of Questionnaire

An anonymous self-administered questionnaire was developed to collect data from pharmacy customers, utilizing both professional networks and the snowballing technique, a method in which existing study participants refer or recruit additional participants from among their acquaintances. The 29-item questionnaire comprises five primary sections: demographics; consumer perceptions regarding the safety and effectiveness of CMs, as well as the reasons for using CMs; experience with label warnings; adverse reactions associated with CMs; as well as the reporting of such reactions. The full questionnaire is provided in Supplementary File S1. Before commencing the survey, a pilot study was conducted to assess the clarity and relevance of the questionnaire items. The primary objectives included evaluating the duration required to complete the survey and identifying potential sources of response bias. Six actively practicing pharmacists participated in this preliminary assessment by engaging with the survey instrument. To ensure diverse response types and efficient completion, a variety of question formats were employed, such as multiple choice, open-ended free text, and Likert scale responses. For queries related to perceptions of CMs’ efficacy, side effects, and safety, respondents used a Likert scale ranging from strongly agree to strongly disagree, where a score of 5 represented strongly agree and 1 denoted strongly disagree.

### 2.5. Data Analysis

**Figure 1:**
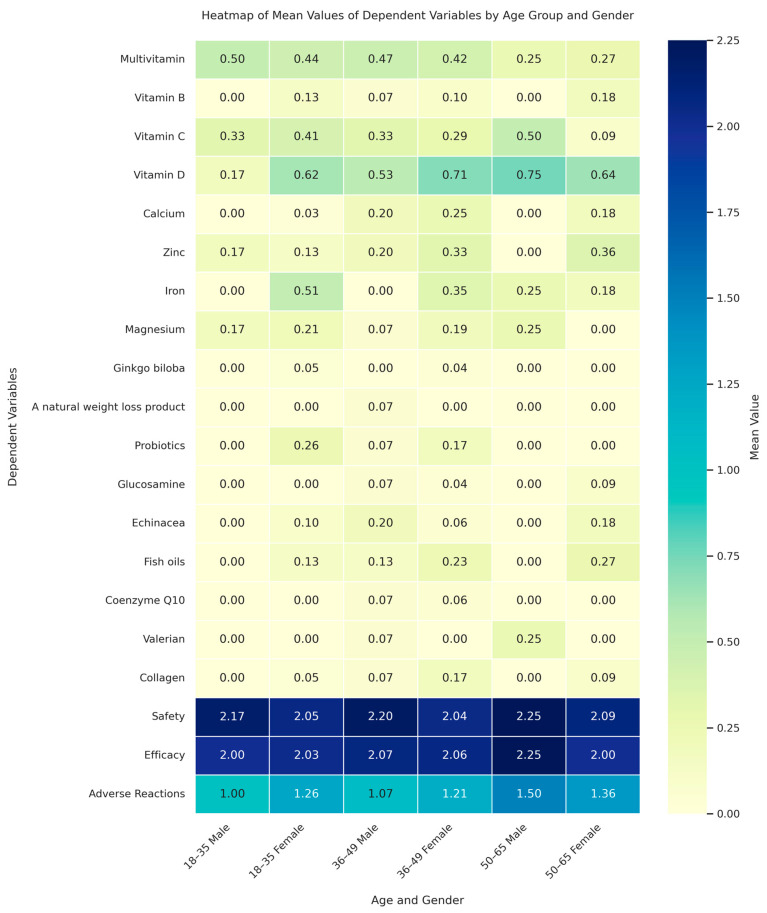
Heatmap of mean values of dependent variables by age group and gender. This heatmap illustrates the average scores of participants concerning their use of various product-based CMs, as well as their perceptions on safety, efficacy, and experienced adverse reactions, segmented by age group and gender.

The collected data were analyzed using IBM Statistical Package for the Social Sciences Software (SPSS, Ver. 28). Descriptive statistics, including frequencies and percentages, were employed to analyze demographic characteristics and variables. Bivariate associations between dependent variables were examined using Pearson’s correlation coefficient. This comprehensive analysis allowed us to derive valuable insights into the relationships among the variables studied. Ordinal logistic regression was utilized to evaluate the influence of individual and grouped CMs on age categories. For the purpose of this analysis, CMs were categorized into three distinct groups: vitamins, which included multivitamins, vitamin B, vitamin C, and vitamin D; minerals, comprising calcium, zinc, iron, and magnesium; and other CMs, which encompassed a range of products, such as Ginkgo biloba, natural weight loss products, probiotics, glucosamine, echinacea, fish oils, coenzyme Q10, St. John’s Wort, valerian, and collagen. Additionally, binary logistic regression was employed to examine gender-based differences in CM usage. We used a multivariate analysis of variance (MANOVA) to investigate how the joint effects of age and gender influence a range of dependent variables, including different types of CMs and perceptions related to their safety, efficacy, and adverse reactions. The independent variables in MANOVA were age groups, divided into three categories: 18–35, 36–49, and 50–65 years, and gender, categorized into male and female. MANOVA was performed using the statsmodels library in Python version 3.9, and the core visualization library matplotlib was utilized to create the heatmap illustrated in Figure 1. A heatmap is a visual representation that uses color to show the magnitude of different values in a matrix format. In our study, the heatmap in Figure 1 displays the average scores of participants on their use of various CMs, as well as their perceptions of safety, efficacy, and adverse reactions, categorized by age group and gender. To create this heatmap, we processed the data to calculate the mean values for each dependent variable. These values were then represented using a gradient color scale, which helps to easily spot differences and patterns across different age and gender groups.

## 3. Results

### 3.1. Demographic Characteristics of the Participants Included in the Study

A total of 180 participants completed the online questionnaire, of which 125 participants responded to having been using product-based CMs obtained from pharmacies and supermarkets. Participants’ demographic characteristics are described in Table 1. All participants were from Melbourne, Australia.

**Figure 2:**
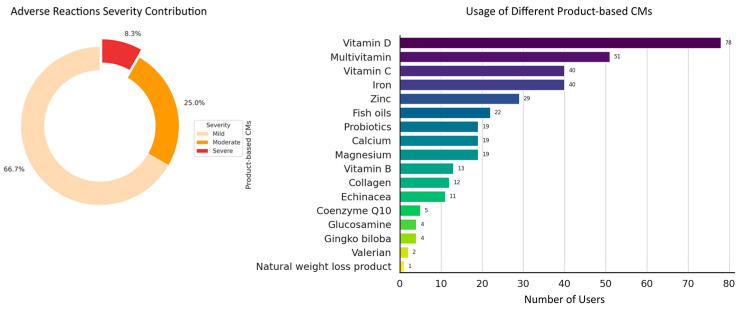
Adverse reactions severity distribution and usage of different product-based CMs. The pie chart (**left**) illustrates the distribution of adverse reaction severities, with 66.7% categorized as mild, 25.0% as moderate, and 8.3% as severe. The bar chart (**right**) shows the usage of different product-based CMs among participants, with vitamin D being the most commonly used (78 participants), followed by multivitamin (51 participants) and vitamin C (40 participants).

The majority of participants were 18 to 50 years of age (84.8%), with the highest percentage (48.8%) belonging to the 31–50-year age group. Females constituted a majority of the participants (79.2%). Education levels were varied, with 76.8% of participants having a university degree, 16% a diploma or certificate, and 7.2% having completed only high school education. A total of 44.8% of participants reported being in very good health, 31.2% indicated they were in good health, 17.6% rated their health as excellent, and 6.4% reported their health as poor. Additionally, 33.6% of participants reported currently taking prescription medicines. Participants used a diverse range of CM products. Vitamin D was the most commonly used CM, with 62.4% of participants reporting its use, followed by multivitamins at 40.8% and both vitamin C and iron at 32% each. In contrast to these more frequently used supplements, only one participant reported using a CM product for weight loss, categorized as a “natural weight loss product”; however, no further details about this product were provided. Figure 2 represents the number of participants using different CMs. The consumers predominantly purchased CM from pharmacies (88%) and supermarkets (24%). In total, 27 participants (21.6%) reported using more than one source to obtain CM products. Table 1 shows the primary sources through which respondents accessed information regarding CMs. Notably, the internet/media emerged as the predominant source, with 37.1% (47) of the total respondents relying on this platform. Doctors were consulted by 30% (37) of respondents, while pharmacists were consulted by 24% (31) of respondents. Nineteen percent (24) of respondents sought advice from family and friends. In contrast, herbalists, the CM labels, and other sources constituted smaller proportions, each representing less than 15% of respondents. Participants aged 65 years and older (n = 4) were excluded from certain statistical analyses due to the small sample size, which limited the statistical power and validity of comparisons involving this subgroup.

### 3.2. Insights into Participants’ Attitudes and Practices Related to CMs

The survey uncovered various reasons why people turn to CMs. For about a third of the participants (32%), their main drive was to maintain good overall health and feel better. One in four (25.6%) leaned towards CMs for preventive care, trying to stay clear of illnesses. Nearly 30% said they chose CMs because it matched their lifestyle, while an equal number relied on recommendations to use CM. A smaller group (12%) sought CMs to take more charge of their health, and 10.4% believed CMs worked just as well as, or better than, regular medicines.

In response to whether label warning statements affected participants’ decision to take a product or prompted them to seek additional information, participants provided the following responses: Seven participants (5.7%) indicated that these warnings stopped them from taking the product. A larger group of 61 participants (48.8%) stated that the warnings led them to seek further information before using the product. On the other hand, 38 participants (30.4%) reported that, despite reading labels, they were not concerned by any warning. Interestingly, 13 participants (10.4%) admitted to not reading labels at all, and 3 participants (2.4%) acknowledged reading labels but facing difficulties in understanding them. The majority (n = 97, 77.6%) expressed a belief in the safety of CMs, while 98 participants (78%) believed in their efficacy.

### 3.3. Adverse Reactions and Responses to CMs

Among the study participants, 18.5% of participants reported encountering adverse reactions. The majority of those who experienced side effects identified the reactions as mild (66.7%), followed by moderate (25%), and a smaller portion as severe (8.3%). Figure 2 illustrates the distribution of adverse reaction severities. Interestingly, most of the participants did not seek help from HCP and self-managed these adverse reactions. Notably, 33.3% ceased the use of the CMs, while 25% decreased their dosage. Only 16.7% of participants sought advice from an HCP after experiencing side effects. Interestingly, communication regarding adverse reactions was more likely to occur with doctors and family/friends (29.2% and 41.7%, respectively), while engagement with pharmacists (4.2%) was less frequent. Participants were prompted with an open-ended question to report any side effects they experienced, if applicable. Several participants mentioned experiencing diarrhea, constipation, bloating, and stomachache.

### 3.4. Correlations Between Key Variables and CMs Consumption

Table 2 presents correlations between key variables and CMs consumption. Notably, a positive correlation emerged between overall health status and the perceived effectiveness of CMs (r = 0.43, *p* < 0.01). Additionally, participants’ perceptions of CMs’ efficacy showed a positive correlation with their reported effectiveness after usage (r = 0.48, *p* < 0.01). The study also highlighted a positive relationship between perceptions of CMs’ quality and their perceived effectiveness (r = 0.41, *p* < 0.01). Furthermore, participants who held the view that CMs were more effective than prescription medications demonstrated a moderate positive correlation with the perceived efficacy of CMs (r = 0.36, *p* < 0.01). These significant associations shed light on the interrelation between health perceptions, beliefs about CMs’ efficacy, quality, and effectiveness, providing insights into participants’ attitudes and beliefs towards CMs’ utility and potency. Additionally, people who used CMs for minor health issues tended to believe that these products were of higher quality (r = 0.19, *p* < 0.05); however, it is important to note that this represents a weak correlation, and the statistical significance may be attributed to the sample size rather than the strength of the relationship.

### 3.5. Demographic Trends in CMs Usage and Perceptions

The MANOVA indicated that age and gender do not generally have a significant impact on the dependent variables (the use of different types of CMs, safety, efficacy, and adverse effects) (*p* > 0.05), according to most test statistics. However, Roy’s greatest root test indicated potential effects of age and gender that might be worth further investigation, yielding a significant p-value for age (*p* = 0.01) and a marginally significant value for gender (*p* = 0.06, Figure 1). The ordinal logistic regression analysis showed a significant association between calcium consumption and higher age groups (coefficient = 1.658, *p* = 0.006), indicating a higher tendency for older adults to use calcium supplements. In the binary logistic regression analysis, a significant gender-based pattern was observed in iron consumption, with females showing higher consumption (coefficient = 2.177, *p* = 0.007).

## 4. Discussion

The findings from this study collectively underscore the multifaceted nature of factors influencing the utilization of product-based CMs available over the counter (e.g., vitamins, minerals, herbal preparations), presenting an understanding of consumer attitudes and practices.

The majority of CM users (48.8%) who participated in this study reported being attentive to product labels, and label warnings in most cases prompted them to seek further information or advice before using the products. This reflects a heightened awareness among consumers of potential safety concerns associated with CMs. A substantial portion of consumers (30.4%) indicated that while they read the product label information, label warnings did not lead them to discontinue use or seek additional information. A smaller fraction (10.4%) reported neither reading nor comprehending the labels (2.4%). The diverse responses received from the study participants highlight the importance of enhancing consumer education and label comprehension to foster safer CMs use practices. It should be noted that the majority of participants in this study held a university degree and that these data may be skewed. Therefore, a large-scale study is warranted to further explore the impact of label warnings and label content on CM usage. However, in line with this recommendation, a study conducted in Serbia underscores the need for educating consumers on how to use information on CMs labels and the establishment of an effective monitoring system for dietary supplements’ labeling on a national scale to protect consumers and their well-being [22].

Previous research also indicates that consumers pay considerable attention to health claims or indications stated on CM labels, as these manufacturer claims aid in establishing the perceived benefits of the product [22]. Research suggests that these health claims play a crucial role in shaping consumer perceptions and influencing purchase decisions [22]. If consumers cannot establish benefits of a product due to inadequate labeling or information, it is likely that this will lead to a loss of sales. Therefore, the TGA aims to implement changes in labeling requirements to ensure that health claims and indications are effectively communicated to consumers, thus supporting informed decision-making and maintaining consumer confidence in CMs [23]. In Australia, most complementary medicines are included in the Australian Register of Therapeutic Goods (ARTG) as listed medicines (AUST L). Listed medicines contain low-risk ingredients and use low-level indications selected from a list that is approved by the TGA. Some complementary medicines may include a “TGA assessed (AUST L(A))” claim, which indicates that the TGA has conducted a pre-market assessment of the efficacy of the medicine’s indications to ensure claims are evidence-based [24].

Another noteworthy finding is that while 54% of participants sought advice from healthcare professionals, primarily doctors and pharmacists, this still leaves nearly half relying on other sources. Given that 88% obtained CMs from pharmacies, the lower engagement with pharmacists (24%) is particularly striking. Previous studies have similarly shown variability in healthcare professional involvement, with some reporting even lower consultation rates among CM users [4,25]. Although most CM users purchase their products from pharmacies, only a fraction of participants sought consultation with pharmacists regarding CMs-related information and adverse effects. It has been noted in this study that nearly 30% of patients do not heed advice despite label warnings or fail to read the label warnings (10%). Lack of disclosure of CM usage with healthcare professionals can also pose a challenge in providing appropriate care for patients in a healthcare setting due to the inability to identify drug interactions and the impact of CM use on treatment outcomes [24]. For example, it has been reported that patients undergoing cancer treatment have a greater propensity to use CMs [26]. In surgical patients, inappropriate CM usage [27] can increase the risk of bleeding and can also lead to interactions with anesthetic agents, changing the pharmacokinetics of anesthetic agents.

The regression analysis offered valuable insights into how different demographics consume CMs. We found that older adults are more likely to use calcium supplements, which makes sense given their need to maintain bone health as they age (coefficient = 1.658, *p* = 0.006). Similarly, the analysis showed that women are more likely to take iron supplements (coefficient = 2.177, *p* = 0.007). This aligns with the well-known fact that women have higher iron requirements due to menstruation [28]. Complementing these findings, the MANOVA results indicated that age and gender do not generally have a significant impact on the dependent variables, with a few exceptions noted. Specifically, Roy’s greatest root test suggested potential effects worth further investigation. This comprehensive approach helped gain a deeper understanding of how different demographic factors influence CMs consumption patterns. It provided valuable insights into trends that could benefit from targeted interventions or further research. For instance, older adults tend to use supplements such as calcium more frequently, indicating a possible increase in CMs usage as people age. Similarly, perceptions of safety and efficacy showed variation, although not significant, often correlating with higher usage rates. These findings highlight the need for further research to explore the differences more deeply. Although a wide range of CMs were reported, weight-loss-related CMs were rarely mentioned, with only one participant indicating such usage. This may reflect underreporting or lack of recognition of weight-loss products, such as CMs. Future studies should specifically investigate the prevalence, types, and safety perceptions surrounding weight-loss CMs.

Additionally, this study highlights a communication gap between consumers and healthcare professionals regarding CMs use. Exploring healthcare professionals’ awareness of patient CM consumption would be a valuable area for future research, as increasing clinician–patient dialogue could enhance safe CMs use. A qualitative study conducted in Ontario, Canada, reported that the majority of consumers are unable to find healthcare providers who could adequately address their inquiries about CMs [25]. This disconnection between perceived roles and the actual utilization of pharmacists’ knowledge and skills presents an opportunity to bridge this gap by promoting pharmacists as dependable sources of CMs information. Drawing on the knowledge of pharmacists to provide evidence-based advice on CMs, prescription drug interactions, and adverse effects, pharmacists are well-positioned to greatly improve the safe use of CMs [4]. However, it is important to note that pharmacists in Australia have expressed a lack of confidence in their knowledge of CMs, which might impede their effective engagement with patients in providing comprehensive information about CMs [29]. Ensuring proper training, continuous education, and access to reliable information on CM products for community pharmacists is essential to instill the confidence needed to approach patients and address their questions and concerns about CMs. This recommendation echoed that of another study highlighting the need to have additional education and training for pharmacists to increase their knowledge and confidence [30].

The analysis of participants’ beliefs regarding CMs effectiveness in comparison to prescription medications is thought-provoking. While a substantial proportion (15.8%) of consumers believed CMs are more effective than prescription medications, almost half (41.6%) perceived CMs as less effective than prescription medications. This contradicts previous findings that highlighted the importance of CMs over prescribed medications [31]. The difference could be attributed to the higher education levels of participants in this study, with all participants in this study having completed high school or tertiary-level education, compared to 72.6% with primary education only and 11.2% with no formal education in the previous study. This finding underscores the role of consumer education level in shaping perceptions of CMs in relation to conventional medications.

The results of this study also shed light on the CM-related adverse reactions experienced by participants. It is notable that 18.5% of participants reported encountering adverse reactions. This finding challenges the common notion that CMs are devoid of side effects, emphasizing the importance of acknowledging potential risks associated with these products [32]. The majority of reported adverse reactions were categorized as mild (66.7%), with moderate reactions accounting for 25% and a smaller proportion classified as severe (8.3%). In a comparable manner, a cross-sectional study conducted in England found that almost 50% of the study participants using CMs reported encountering adverse events [12]. These adverse effects were primarily of a mild to moderate nature, with only a few serious or life-threatening [12]. This contradiction highlights the necessity for comprehensive and accurate information dissemination, robust communication channels, and increased consultations with healthcare professionals to ensure the safe use of CMs by consumers [33,34].

Interestingly, participants in this study demonstrated a trend towards self-management when faced with adverse reactions. It is noteworthy that 33.3% of participants discontinued the use of the CM product upon encountering adverse reactions, while 25% chose to decrease their dosage. This inclination towards self-adjustment could indicate that consumers perceive themselves as the primary decision-makers in managing their health-related experiences. At the same time, it raises questions about the adequacy of information provided to consumers about potential side effects and their appropriate responses. Additionally, the study reveals that participants gather information about CMs from various sources, with the internet and media playing a significant role. Alarmingly, 37.1% of respondents relied on the internet for information. These findings raise concerns regarding the quality of CM information available online. A previous review of Wikipedia entries on popular herbal supplements found significant gaps in crucial information, including details on drug interactions, effects during pregnancy, and contraindications [33]. Moreover, the writing level of the entries exceeded the recommended comprehension level for readers [35]. Additionally, 13 websites related to common herbal supplements lacked important safety information and failed to recommend consulting healthcare professionals before using the products [35]. These findings underscore the potential risks associated with relying solely on online sources for CMs-related information. These findings align with a previous study conducted in the Czech Republic, highlighting the impact of mass media and family traditions [36]. This underscores the influence of media in shaping individuals’ perceptions and decisions related to CMs, emphasizing the need for a comprehensive understanding of the interplay between media, consumer behavior, and CMs usage [37]. In addition, this highlights the need for healthcare professionals to play a more active role in guiding consumers due to the potential risks associated with inappropriate CMs use.

### Limitations and Future Directions

While this study provides valuable insights, several limitations should be acknowledged. The reliance on self-reported data and a cross-sectional design limits the establishment of causal relationships. Additionally, the relatively small sample size and the fact that most participants held a university degree and were from a specific geographic region may impact the generalizability of the findings. This distribution may have been influenced by the fact that students were responsible for collecting the data, potentially resulting in a sample biased towards individuals within their academic circles. The underrepresentation of participants aged over 65 could also be a consequence of this sampling method. Furthermore, the higher proportion of vitamin and mineral users in our sample may limit the generalizability of our findings to broader product-based CMs usage patterns. In addition, disease-state information (e.g., presence of chronic illness or cancer) was not collected, limiting our ability to explore potential interactions between CMs and medical conditions. Cultural and social determinants of CMs use were also not comprehensively captured, which limits insights into how these factors shape consumer decisions and beliefs. Future studies should consider integrating these aspects using qualitative or mixed-methods approaches. The survey did not include the internet as a listed source for purchasing CM products, which may have contributed to underreporting of online purchases. Furthermore, although an ‘Other’ category was provided, participants may not have considered listing online sources there. Additionally, while this study assessed whether label warnings influenced consumer behavior, it did not explore which specific types of warnings (e.g., drug interactions, organ toxicity, and allergy risk) were most impactful—this represents an opportunity for more detailed future research. Nevertheless, this study highlighted the need for further research to validate these insights. Moreover, qualitative research could offer a deeper understanding of participants’ motivations, barriers, and experiences related to CMs usage, label warnings, and pharmacist consultation. Future studies should also focus on the use of CMs for specific purposes, such as weight loss, and examine the extent to which these are reported or discussed with healthcare professionals.

## 5. Conclusions

In conclusion, this study examined various facets of CMs usage, including label warnings and consumer beliefs, and contributes to a better understanding of CMs consumption patterns. The findings emphasize the need for targeted efforts to improve label comprehension, promote pharmacist consultation, and enhance consumer education about CMs. Ultimately, fostering informed decision-making in CMs usage can contribute to safer practices and better outcomes for consumers. This study also highlights the need for larger studies to gather more information on CMs label usage and appropriateness.

## Figures and Tables

**Table 1:** Demographic characteristics of study participants (n = 125).

Variables		Number of Participants	Percentage %
Age	18–30	45	36.0
	31–50	61	48.8
	51–65	15	12.0
	65+	4	3.2
Sex	Men	26	20.8
	Women	99	79.2
	Prefer not to specify	0	0.0
Education	High school	9	7.2
	Diploma/certificate	19	16.0
	University	96	76.8
Overall health status	Excellent	22	17.6
	Very good	56	44.8
	Good	39	31.2
	Poor	8	6.4
Currently taking any prescription medicines	Yes	42	33.6
	No	83	66.4
Specify CMs you are using or have used in the past six months	Multivitamin	51	40.8
	Vitamin B	13	10.4
	Vitamin C	40	32.0
	Vitamin D	78	62.4
	Calcium	19	15.2
	Zinc	29	23.2
	Iron	40	32.0
	Magnesium	19	15.2
	Gingko biloba	4	3.2
	Natural weight loss product	1	0.8
	Probiotics	19	15.2
	Glucosamine	4	3.2
	Fish oils	22	17.6
	Echinacea	11	8.8
	Coenzyme Q10	5	4
	St. John’s Wort	0	0
	Valerian	2	1.6
	Collagen	12	9.6
	Other	14	11.2
Source of CMs	Pharmacy	110	88
	Supermarket	30	24
	Natural supplements shops	10	8
	Others	2	1.6
Source of CM information	Family/friends	24	18.8
	Internet/media	47	37.1
	Herbalist	13	10.2
	Label of medicine	15	12.2
	Pharmacists	31	24.3
	Doctors	37	29.8
	Others	2	1.6

**Table 2:** Correlations between key variables and product-based CMs consumption.

	Overall Health Status	Reported CMs Effectiveness	Perceptions of CMs Safety	Perceptions of CMs Efficacy	Perceptions of CMs Side Effects	Perceptions That CMs More Effective than Medications	Perceptions of the Good Quality of CMs	Perceptions of Better Usage of CMs for Minor Ailment Instead of Medications
Reported CMs effectiveness	0.165	1						
Perceptions of CMs safety	0.02	0.03	1					
Perceptions of CMs efficacy	0.073	0.143	0.48 **	1				
Side effects	−0.051	0.154	−0.096	−0.092	1			
Perceptions that CMs more effective than medications	−0.001	−0.187 *	−0.076	−0.066	0.36 **	1		
Perceptions of the good quality of CMs	−0.013	−0.125	−0.117	−0.023	0.19 *	0.41 **	1	
Perceptions of better usage of CMs for minor ailment instead of medications	−0.115	−0.246 **	0.004	−0.048	0.08	0.38 **	0.19 *	1

*p*-values derived from Pearson’s correlation: * *p* < 0.05 and ** *p* < 0.01.

## Data Availability

The data underlying this study are not publicly available due to privacy and ethical restrictions but may be accessed upon reasonable request to the corresponding author, subject to institutional and ethical guidelines ensuring participant confidentiality.

## Supplementary Materials

The following supporting information can be downloaded at https://www.mdpi.com/article/10.3390/pharmacy13030061/s1. The Supplementary File S1 provided is the survey questionnaire used in this study. It contains all the items and response options administered to participants.
