# Supplementary material for: Exploring Complementary Medicine Usage, Consumer Perceptions, and Impact of Label Warnings: A Cross-Sectional Study in Melbourne, Australia
Source: Pharmacy (Basel). 2025 Apr 27;13(3):61. doi: 10.3390/pharmacy13030061 (PMC12101403; doi:10.3390/pharmacy13030061)
Supplement: Supplementary file 1 [file pharmacy-13-00061-s001.zip › pharmacy-3536967-supplementary.pdf]

English

Default Question Block

Are you currently taking or have ever taken any complementary and alternative medicines? Examples of these products include herbal medicines, nutritional supplements, vitamins and minerals.

- Yes
- No

When did you last take complementary or alternative medicines?

- 1-3 months ago
- 4-6 months ago
- More than 7 months ago
- Never

What is your age?

- 18-30
- 31-50
- 51-65
- 65+

What is your gender?

- Male
- Female
- Prefer not to specify

What is your highest level of education?

- Primary school
- High school
- Diploma/certificate
- University

How would you describe your overall health?

- Excellent
- Very good
- Good

- ◯ Poor

Please indicate if you have any of the following medical conditions

- ☐ Arthritis
- ☐ Asthma
- ☐ Back pain
- ☐ Cardiovascular disease
- ☐ Diabetes
- ☐ Mental health conditions
- ☐ Cancer
- ☐ Chronic obstructive pulmonary disease
- ☐ Liver impairment
- ☐ I don't have any medical conditions
- ☐ Other, please specify: ______

Are you currently taking any prescription medications?

- ◯ No
- ◯ Yes

Please specify the complementary and alternative medicines you are currently using or have used in the past

- ☐ Multivitamin
- ☐ Vitamin B
- ☐ Vitamin C
- ☐ Vitamin D
- ☐ Calcium
- ☐ Zinc
- ☐ Iron
- ☐ Magnesium
- ☐ Ginkgo biloba
- ☐ A natural weight loss product
- ☐ Probiotics
- ☐ Glucosamine
- ☐ Echinacea
- ☐ Fish oils
- ☐ Coenzyme Q10
- ☐ St John's Wort
- ☐ Valerian
- ☐ Collagen

- ☐ Other, please specify: _____

From where do you normally obtain your CAMs?

- ☐ Supermarket
- ☐ Pharmacy
- ☐ Herbal/Natural product shop
- ☐ Other, please specify: _____

What are the reasons for using the complementary and alternative medicines mentioned

- ☐ It keeps me healthy and gives me a sense of wellbeing
- ☐ To prevent disease
- ☐ To treat a specific disease or symptom
- ☐ It fits into my way of life
- ☐ My health problem is not serious enough to take prescription medicines
- ☐ They work better or just as well as other medicines
- ☐ They were recommended to me
- ☐ It gives me a sense of control over my health
- ☐ Other, please specify: _____

How effective are the complementary and alternative medicines you take?

- ○ Very effective
- ○ Moderately effective
- ○ Slightly effective
- ○ Not effective
- ○ Don’t know/unsure

Where do you mainly find out information about complementary and alternative medicines?

- ☐ Family/Friends
- ☐ Internet/Media
- ☐ Naturopath/Herbalist
- ☐ Label of medicine
- ☐ Pharmacist
- ☐ Medical doctor
- ☐ Other, please specify: _____

Has a label warning statement stopped you from taking a product, or led you to seek further information before taking?

- ◯ Yes, it stopped me from taking. What was the label warning about?
- ◯ Yes, it led me to seek further information
- ◯ No, I don't read labels
- ◯ No, I read labels but have not been concerned by any warnings
- ◯ No, I read labels but don't understand them
- ◯ Other, please specify:

Have you experienced any adverse reactions (unwanted effects such as rash, nausea, diarrhoea, etc.) to complementary and alternative medicines?

- ◯ No
- ◯ Yes
- ◯ Unsure

How serious was the reaction?

- ◯ Severe (required hospitalisation)
- ◯ Moderate (symptoms required a visit to a doctor or other healthcare professional)
- ◯ Mild (no specific treatment was required)

Please specify the adverse reaction/s you experienced

What did you do about the reaction?

- ◯ I stopped using the CAM product
- ◯ I reduced the dose I was taking
- ◯ I changed to another CAM product
- ◯ I sought advice from a healthcare professional
- ◯ Other, please specify:

Who did you tell about the adverse reaction?

- ◯ I didn't tell anyone
- ◯ Family/Friend
- ◯ Naturopath/Herbalist
- ◯ Product manufacturer
- ◯ A pharmacist

- A doctor
- Other, please specify:

What were some barriers for you in reporting your adverse reaction?

Do you believe complementary and alternative medicines are safe to use?

- No
- Yes
- Other, please specify:

Do you believe complementary and alternative medicines are effective for you?

- No
- Yes
- Other, please specify:

Please select the most suitable option for the following questions:

Complementary and alternative medicines are safer than prescription medications

- Strongly disagree
- Disagree
- Neutral
- Agree
- Strongly agree

Complementary and alternative medicines are more effective than prescription medications

- Strongly disagree
- Disagree
- Neutral
- Agree
- Strongly agree

In general, complementary and alternative medicines are of good quality

- Strongly disagree
- Disagree
- Neutral

- Agree
- Strongly agree

Complementary and alternative medicines generally do not have side effects

- Strongly disagree
- Disagree
- Neutral
- Agree
- Strongly agree

Doctors, nurses, and pharmacists should recommend complementary and alternative medicines more often

- Strongly disagree
- Disagree
- Neutral
- Agree
- Strongly agree

For chronic medical conditions (e.g., high blood pressure, diabetes, high cholesterol), I would prefer to take complementary and alternative medicines rather than a prescription medication

- Strongly disagree
- Disagree
- Neutral
- Agree
- Strongly agree

For minor ailments (e.g., cough due to a cold, indigestion, aches and pains), I would prefer to take complementary and alternative medicines than a prescription medication

- Strongly disagree
- Disagree
- Neutral
- Agree
- Strongly agree

Please add your name and number for contact purposes if you wish to go into the draw to win $20 voucher

Name

Number
